# Barriers to accessing malaria treatment amongst school-age children in rural Malawi

**DOI:** 10.1186/s12936-023-04695-z

**Published:** 2023-09-06

**Authors:** Patani Mhango, Monica Patricia Malata, Effie Chipeta, Alick Sixpence, Terrie E. Taylor, Mark L. Wilson, Lauren M. Cohee, Charles Mangani, Don P. Mathanga

**Affiliations:** 1 Centre for Reproductive Health (CRH), Kamuzu University of Health Sciences (KUHeS), Private Bag 360, Chichiri, Blantyre 3, Malawi; 2 School of Global and Public Health, Kamuzu University of Health Sciences (KUHeS), Private Bag 360, Chichiri, Blantyre 3, Malawi; 3 Malaria Alert Centre (MAC), Kamuzu University of Health Sciences (KUHeS), Private Bag 360, Chichiri, Blantyre 3, Malawi; 4 College of Osteopathic Medicine, Michigan State University, East Lansing, MI USA; 5 Department of Epidemiology, School of Public Health, University of Michigan, Ann Arbor, MI USA; 6 Center for Vaccine Development and Global Health, University of Maryland School of Medicine, Baltimore, MD 21201 USA

**Keywords:** School-age children, Case management, Health care access, Universal health care

## Abstract

**Background:**

Over the last two decades, many countries have moved from malaria control toward malaria elimination. However, some sub-Saharan African countries, like Malawi, have recently seen a reversal in malaria control progress with reported increases in confirmed malaria cases. This may be the result of inadequate access to effective malaria control interventions by key population groups that perpetuate transmission. This study aimed to assess the barriers to accessing malaria treatment among school-aged children (SAC) in Malawi.

**Methods:**

A qualitative study was conducted between September and October 2020, where data were gathered in rural Malawi using free-listing interviews, key-informant interviews, semi-structured interviews and focus group discussions. Purposively sampled participants included SAC, parents of SAC, health workers and key stakeholders at community and district levels. Interviews were digitally recorded and transcribed verbatim. Data were organized using NVivo 12 software and analysed using the thematic method.

**Results:**

The study recruited 252 participants, with 156 being SAC, equally divided between boys and girls. Health system barriers to malaria treatment included long waiting hours and queues at clinics, frequent stock-outs of medical supplies, and travel time to the facility. Provider barriers included negative attitude and limited service hours. Individual and cultural barriers included fear of malaria tests and beliefs associating witchcraft as the best treatment for malaria. In addition, COVID-19-related barriers included the inability to follow preventive measures, a shift in focus from malaria to COVID-19, and fear of contracting COVID-19 and/or being tested for COVID-19 at the facility.

**Conclusions:**

This study shows most of the barriers to accessing malaria treatment among SAC are similar to those experienced by other population groups. Furthermore, COVID-19 adversely affected SAC’s access to treatment. Interventions that support SAC access to prompt diagnosis and treatment are urgently needed to improve the effective control of malaria.

## Background

Malaria remains a major public health problem, with an estimated 241 million cases and 627,000 deaths globally in 2020 [1]. In the last two decades, there has been a considerable reduction in malaria burden in many countries [2], mainly due to the expanded use of long-lasting insecticidal nets (LLINs), improved diagnosis using malaria rapid diagnostic tests (RDTs), and prompt treatment with artemisinin-based combination therapy (ACT) [3, 4]. Despite the widespread use of these effective interventions, some sub-Saharan Africa (SSA) countries continue to face a high burden of malaria, with recent reports documenting either stalled progress or increasing annual malaria incidence [1].

Malawi’s progress in malaria control has reversed, with a 37% increase in incidence from 5.2 million cases in 2019 to 7.2 million in 2020 [1]. Factors hypothesized to have contributed to the increasing malaria burden include widespread insecticide resistance of *Anopheles* vector mosquitoes [5, 6], and limited adoption of effective interventions by key population groups, such as school-age children (SAC). In Malawi, as in other malaria-endemic countries, SAC have a higher prevalence of *Plasmodium* infection than other age groups [7–11], are less likely to access treatment [12] and to use LLINs [8, 13–15]. Increasing evidence suggests that SAC are also a key reservoir of *Plasmodium* transmission [11, 12, 16, 17]. Thus, the high infection prevalence and the low access to key interventions among SAC is a cause for concern.

Malawi's National Malaria Control Programme (NMCP) treatment guidelines require individuals with malaria-like symptoms get a malaria rapid diagnostic test (RDT) before receiving first-line anti-malarial treatment [18]. Traditional beliefs, stock-out of anti-malarial drugs, long queues, and long distances to facilities have been cited as reasons for limited access to malaria diagnosis and treatment of pregnant women and/or children under five years old [19, 20]. However, no previous studies have explored barriers to access for SAC, despite this group having the highest malaria infection prevalence and the lowest access to diagnosis and treatment in Malawi. In this study, qualitative methods were used to explore barriers to malaria treatment among SAC in southern Malawi.

## Methods

### Study design and setting

This qualitative study was nested within a larger cohort study evaluating the effectiveness of LLINs in reducing *Plasmodium* infection and malaria disease in Malawi. The study was conducted in two rural districts in southern Malawi, Machinga and Balaka, where malaria transmission is intense and year-round, peaking during the rainy season (December to April). Despite the scale-up of malaria interventions, such as malaria diagnosis and treatment, LLINs and indoor residual spraying (IRS), reductions in malaria risk and morbidity have been modest. In 2020, the prevalence of *Plasmodium falciparum* infection remained high (≥ 25%) in most areas of these districts, with Machinga (pop. 713,000) and Balaka (pop. 477,000) recording 212,489 and 160,598 confirmed malaria cases, respectively. Universal malaria interventions, such as LLINs, access to prompt diagnosis and treatment, and intermittent preventive treatment in pregnancy (IPTp) are provided in the two districts. In 2018, a mass distribution campaign of ITNs was conducted in these districts, with a target of one ITN for every two people.

Nearly half of the population is < 15 years of age, which is the group that experiences the greatest malaria burden [7]. In 2021, a SAC cohort had a *P. falciparum* prevalence of 38% (Cohee, pers. commun.). Currently, there are no specific malaria interventions targeting SAC, as they are also covered by universal interventions. Primary school education is free and school enrolment for children aged 6–15 years is generally high.

Due to the elevated prevalence of malaria in the two districts, the Malawi International Center of Excellence in Malaria Research (ICEMR) is conducting studies to understand why and how malaria remains intransigent in the region. The on-going research projects combine measurement of *Anopheles* vector abundance and behaviour, *Plasmodium* infections of vectors and humans, and behavioural risk factors for transmission and disease. Several longitudinal cohorts are being followed to understand both population-level factors influencing malaria prevention and control interventions, and individual-level host and parasite contributions that influence the spectrum of outcomes, ranging from asymptomatic infection to uncomplicated illness to severe malaria.

### Study population and sampling

A 2018 census identified and geo-located all villages and households in the study area, aiding in the selection of a primary school within 5 km of the nearest health facility in both districts. SAC, parents/guardians of SAC, community leaders, teachers, health workers and members of the district health management team (DHMT) were invited to participate in focus group discussions (FGD) and/or individual interviews. Prior to participation, written parental consent and assent from SAC, as well as individual written consent for the adults, were obtained.

Maximum variation purposive sampling was used to select individual SAC aged 10–18 years, parents/guardians of SAC aged 5–18 years, and key stakeholders at the community and district levels. Ten SAC (10–18 years old) were randomly selected from each upper primary school classes (standard 6–8) using a class register. Parents/guardians of some selected SAC and those not selected were invited to participate, and another set of parents/guardians of non-school-going children were also conveniently sampled from the village to validate and generate consensus views.

The study utilized a range of qualitative methods to gather data from different participant groups, including Free Listing Interviews (FLIs), Focus Group Discussions (FGDs), Key Informant Interviews (KIIs) and semi-structured interviews (SSIs). FLIs were used to collect initial qualitative data from SAC participants, including people’s views of particular health-related issues [21]. FGDs provided an opportunity for in-depth exploration of SAC (10–18) and their parents/guardians regarding access to malaria treatment. KIIs with District Health Management Teams (DHMTs) and SSIs of health workers were used to obtain feedback on barriers to treatment.

### Data collection

FGDs for SAC were conducted at the school premises, after all teachers and other learners had left the school for the day, so that nobody could observe the discussions. FGDs for parents/guardians and KIIs for community leaders were conducted in the community at a convenient and private setting. FGDs were comprised of eight participants each, aggregated by gender, with FGDs involving SAC also being aggregated by class (grade). FGDs and interviews with SAC, parents/guardians and community leaders were conducted in Chichewa, the local language. Interviews with teachers, DHMT members, and health workers were conducted in a language of their choice (Chichewa or English). Each FGD was facilitated by an experienced and trained research assistant who had a good command of the local language. A note taker audio-recorded the discussions and made hand-written notes. FGDs, KIIs and SSIs lasted for 1–1.5 h.

### Data management and analysis

All FGDs, KIIs and SSIs were audio-recorded, transcribed verbatim, and translated into English (if necessary) by trained research assistants. All field notes taken during the interview were written in greater detail immediately after the interview or FGD. Thematic analysis method, combining deductive and inductive approaches, was employed to analyse the data. A codebook was developed following the a priori codes, with new codes added as the analysis progressed. A qualitative software data package (NVivo version 12) was used to organize and manage the data. All transcripts were double-coded by three individuals, with periodic checks for inter-coder discrepancies. Clear coding guidelines were established collaboratively before coding began, to manage differences in opinion. These guidelines reduced disagreements and ensured coding consistency. Frequent meetings were held to review the coding, discuss different perspectives, and reach resolutions. This involved revisiting guidelines, clarifying codes, and examining data for deeper insights. After coding, the analysis team familiarized themselves with the data and searched for patterns in the codes to determine themes. Standard data reduction techniques were used to meticulously examine codes, searching for sub-themes and patterns across the transcripts. The themes were then reviewed, identified for subthemes and summarized.

### Ethics

The study was reviewed and approved by the Malawi College of Medicine Research Ethics Committee (P.03/20/2974) and the Michigan State University Biomedical and Health Institutional Review Board (STUDY00004251).

## Results

A total of 252 study participants were recruited, of which 156 (62%) were SAC, equally divided between boys and girls (Table 1).

**Table 1:** Study population by participant type

Data collection method	Population group	No. of interviews or FGDs	No. of participants from Machinga district	No. of participants from Balaka district	Total no. of participants
Free listing interviews (FLI)	SAC	60	30	30	60
Focus group discussions (FGD)	SAC	12	48	48	96
Focus group discussions (FGD)	Parents or guardians	8	32	32	64
Key informant interviews (KII)	Community leaders and teachers	12	6	6	12
Key informant interviews (KII)	District Health Management Team Members	8	4	4	8
Semi-structured interviews (SSI)	Health workers	12	6	6	12
Total		112	126	126	252

Four main themes that represented barriers to access to malaria treatment among SAC were identified. They involved the health system, health care providers, individual and cultural factors, and COVID-19 related barriers (Table 2).

**Table 2:** Summary of barriers to accessing malaria treatment for SAC

Major themes	Sub-themes
1. Health system barriers	a. Long waiting hours and queues
	b. Frequent stock-outs of medical supplies
	c. Travel time to get to the facilities
2. Provider barriers	a. Healthcare provider attitude
	b. Service hours not convenient for SAC
3. Individual and cultural barriers	a. Fear of malaria test
	b. Beliefs associating witchcraft as the best treatment for malaria
4. COVID-19 barrier	a. Inability to follow preventive measures
	b. Shift in focus from malaria to COVID-19 c. Fear of contracting COVID-19 and/or being tested for COVID-19 at the facility

### Health system barriers

SAC and their parents/guardians reported that some health system factors acted as barriers to accessing malaria treatment. These included long waiting hours or long queues at the facility, frequent stock-outs of medical supplies, and long distances or travel time to get to the facilities.

#### Long waiting hours and queues

SAC mentioned during the FLIs and FGDs that long waiting times and queues were a barrier to accessing malaria treatment at the health facilities. Even when the SAC was sick, the facilities served the people on a first-come, first-served basis.

“It is very difficult because in this community, we only have one health facility and there are always so many people that we take much time to access the services at the hospital.” FGD_Balaka; Standard 8 Girls.

"The other challenge we face is that there are always long queues at the hospital hence a long waiting time. This is also a barrier to us." (FGD_Machinga; Standard 8 Boys).

“When we go to the hospital, they attend to you late and no proper order is there as they say the one who got there early gets treatment first instead of differentiating that this is a school child and should be attended to first.” (FGD_Balaka; Standard 8 Boys).

Parents/guardians agreed with the SAC that there are always long queues at the facility and attributed this to the fact that the health facilities serve a huge catchment population.

“At our health centre, there are always long queues because they help so many people and you have to wait for a long period of time in order to get the treatment for your child. Sometimes the condition of the child reaches its severe stages just because of the time you spend on long queues waiting to access the treatment”. (FGD_Machinga; Female Parent of SAC).

However, the SAC highlighted that they are at times exempted from standing in the queues when they go to the hospital with a letter from the school authorities. They reported that they are required to be in school uniform and also carry a letter from the head-teacher requesting that the child be assisted urgently. With this, they are given priority to access the services enabling them to promptly return to school.

“When we are sick and if you want to get treatment quickly at the hospital, then you better get a letter from school and wear a [school] uniform, if you do not wear a uniform then you do not receive treatment quickly, you go back they tell you to stand on the line [queue] if not then you have to go back”. (FGD_Balaka Standard 7 Girls).

“It sometimes happens that you are in a school uniform but you do not have a letter from the teacher, they then send you back to get a letter from your school teacher. They write you a letter and you show the doctor that letter and the doctor assist you, only when you have a letter from the teacher it is when you are assisted as a student”. (FGD_Machinga Standard 7 Boys)**.**

“It always becomes a challenge when you visit the health facility without a letter from the school. You are not given any priority at the hospital, only when you go to school and get a letter, be in school uniform then it is when you will be assisted quickly.” (FGD_Machinga Standard 6 Boys).

Despite the exemptions for SAC who reported to the health facility with support letters from the school and dressed in a school uniform, and carrying their "Health Passport", access to care can still be delayed (A Health Passport is a booklet that contains an individual's health information, such as vaccination history, medical conditions, allergies, consultation notes and prescriptions, and other health-related details. It is intended to be carried by the individual and presented to the health care provider whenever seeking care at the health facility). Urgent access to services often depends on how seriously sick they are. When the health care providers saw that the child was not severely ill, they sent them back to join the queue. Other people waiting in the queue at times expressed anger if they saw that students receiving priority services, regardless of their school uniform and accompanying letters. As a result, some SAC just give up and leave without accessing the services.

“When you get the health passport and you get the letter from school too and you find that there are more patients at the hospital and you do not seem to be seriously sick, you are sent to be on the queue.” (FGD_Balaka Standard 7 Boys).

“Even when you get to the facility with the letter and in school uniform, some people you find on the queue at the facility shout at you saying you are not the only person who is sick.” (FGD_Balaka Standard 8 Boys).

#### Frequent stock-outs of medical supplies

Lack of medication at the health facilities was mentioned as one of the barriers to accessing malaria treatment by both parents and SAC. It was reported that SAC sometimes visited the health facilities to access treatment, but were told to buy the medicines from private facilities or pharmacies. However, due to economic challenges, families were not always able to buy the medicines.

“It sometimes happens that the results from malaria test are positive and when you go to get the drugs, you are told that LA [Artemether-Lumefantrine] is not in stock and you need to go and buy yet you do not have money to purchase. They only give you Panadol [acetaminophen].” (FGD_Balaka Standard 8 Girls).

“When you go to the hospital, they tell you that they do not have anti-malaria drugs and you have to buy but most of the times parents do not have the money to buy us the medicine.” (FGD_Balaka Standard 7 Boys).

It was also reported by the SAC that in some cases, the anti-malaria drugs may be in stock but the rapid diagnostic (RDT) kits may be out of stock. As a result, although a SAC may have presented with all the malaria signs and symptoms, they were unable to access treatment due to lack of a positive RDT.

"Sometimes they tell us that there are no testing kits, while medicines are readily available at that moment. So they cannot give you malaria treatment since they did not test for malaria. So they are not sure if it is malaria. We are just told that we should come back on another day because as of now they do not have malaria test kits but maybe on the other day we will find that they have been delivered." (FGD_Machinga Std 8 Girls).

#### Travel time to get to the facilities

The SAC also reported that the long distance between their homes and the health facilities was particularly challenging. Some facilities have large catchment areas, which resulted in people having to travel long distances for treatment. The challenge was amplified when the patient’s condition was critical, and required transportation to the facility. Due to these long distances, community members at times opted to just buy medications from the shops.

“It [the health facility] is far and they do not have any means of transport and they are like aah! I will just buy medication.” (FGD_Balaka Standard 7 Girls).

“Some other children might be living far from the hospital and that their parents might not be able to take them to the hospital; to escort them to the hospital, and for that reason, they just stay [without accessing malaria treatment].” (FGD_Machinga Standard 6 Girls).

“Long-distance to the health facility and the presence of rivers makes it difficult for children in such locations to access health services during the rainy season as most rivers over flood and children fail to cross”. (FGD_Balaka Standard 6 Boys).

### Provider barriers

Some SAC reported that their access to malaria treatment was impeded by factors related to providers such as provider attitude and service hours not being convenient to SAC.

#### Healthcare provider attitude

SAC described that poor health care provider attitudes acted as a barrier to accessing treatment at the facilities. It was reported that some providers were rude and did not treat the patients with the respect they deserved. The providers became irritated when SAC presented a torn health passport.

"Health provider attitude is another problem we face. They don’t care about the sick and they are always harsh when delivering the services." (FGD_Machinga Std 8 boys).

"We are always worried that they will shout at us when we seek health services. So you are always worried when going to a health facility. I heard that some doctors throw away health passports whenever you are failing to explain your problem." (FGD_Balaka Standard 6 Girls).

In some cases, students became ill while at school, but when they brought their school letter to the health facility, providers refused to see them if they did not also have their health passport. This sometimes happened because the facility was close to the school, while their homes were far away, making it difficult to go home and collect their health passport.

“When it happens that you fall ill while at school and you do not have your health passport with you, the teachers write you a letter but when you get there at the hospital, they deny you the services and you have to go back home.” (FGD_Balaka Standard 7 Boys).

Parents concurred with the SAC that health workers were at times unapproachable and unwilling to assist irrespective of the severity of the situation. In some cases, patients were sent back home and told to come the next day for the service, even when the facility was officially open for service, and regardless of the patient’s condition.

“We go to the hospital early so that we can access treatment faster. However, when we get there we find that the doctors come in at their own time, for instance, instead of them opening the hospital early, they show up at 9 o’clock. Sometimes we seek services at night because the child might get sick at any other time, but when we ask for the doctor they tell you that the doctor is not available.” (FGD_Machinga Female Parent of SAC).

“These doctors don’t really help if we come with the sick child at night. They tell us to go back to our homes … even without considering the seriousness of the child's condition.” (FGD_Machinga Male Parent of SAC).

#### Service hours not convenient for SAC

Many SAC reported that delayed opening and limited service hours of health facilities represented a major barrier. It was conveyed that the health care providers reported late for duties, which resulted in SAC staying longer at the health facility and missing school.

"Hospitals sometimes open late and we stay long at the hospital and cannot go back to school." (FGD_Machinga; Standard 8 boys).

### Individual and cultural barriers

The SAC and providers perceived that individual and cultural factors played a role in preventing SAC from accessing malaria treatment. These included fear of malaria test and beliefs associating witchcraft as best treatment for malaria.

#### Fear of malaria test

The SAC reported that some of them avoided malaria treatment due to the fear of the pain of the needle when they are drawing blood samples for malaria tests and the injections when receiving treatment. It was reported that some felt that they were already in pain due to malaria and couldn’t tolerate additional pain from the needles.

“We always get worried about the pain of the injection. You are already in pain and when you think of getting injected, it becomes a concern.” (FGD_Balaka Standard 6 Girls).

“What troubles me is that when I get to the hospital, I should be injected in a painful way.” (FGD_Balaka Standard 7 Boys).

Parents also expressed fears about their blood samples being used not only for malaria screening, but also for other diseases such as HIV. This made them reluctant to allow their children to go to health facilities for malaria treatment, fearing that they would be subjected to tests for which they were unprepared.

"Some people say that the blood sample that they provide is also used to test other diseases such as HIV. As such, people prefer not to come to access malaria treatment because they do not want to be drawn blood which they think would be used for purposes other than malaria tests." (FGD_Balaka Male Parent of SAC).

#### Beliefs associating witchcraft as the best treatment for malaria

For others, traditional beliefs involving witchcraft to treat malaria kept them from using modern heath facilities. SAC are required to have parental support to receive health facility care, and some parents who held traditional beliefs did not allow their SAC to seek care. Some parents initially took their SAC to a witch doctor, and if the child was deemed bewitched, they no longer considered seeking care from a health facility.

“Others go to the witch doctors who tell them that they have been bewitched. Mostly these are the people that do not go to the hospital to get tested. As a result, they just stay without going to access the required treatment at the health facilities.” (FGD_Machinga Standard 8 Girls).

Parents also agreed with the SAC that sometimes people do not accept malaria treatment and prefer to seek traditional medicines even in severe malaria cases.

“In one case of severe malaria where a child was even unconscious, the grandmother stopped me from providing anti-malaria injection because she believed the child was suffering from ‘Kambanga’. She told us that the medicine for the condition is not the injection but traditional medicines found in the villages.” [Kambanga is a term referring to a disease that causes a child to lose consciousness] (SSI_Machinga Service Provider).

### COVID-19 barriers

Participants, including SAC, parents/guardians and community stakeholders, reported that factors related to COVID-19 further hindered SAC’s access to malaria treatment and prevention. These factors included the inability to follow COVID-19 preventive measures, a shift in focus from malaria to COVID-19, and fear of contracting and undergoingCOVID-19 test at the facility.

#### Inability to follow preventive measures

Participants observed that COVID-19 preventive measures, such as the requirement to wear face masks, hindered access to services. SAC who did not own masks were unable to access malaria treatment services in the facilities. Additionally, the stock-out of medical supplies and drugs in the facilities was attributed to the impact of COVID-19.

“The introduction of the rule where those without face masks should not enter hospitals has resulted in those people who find difficulties in breathing when putting on a mask at risk of not getting health assistance when they fall sick.” (FGD_Balaka Standard 8 Girls).

“There are some people who cannot afford to buy face masks so they are told to go back without accessing the services.” (FGD_Machinga Standard 7 Boys).

“To access health services is also a challenge with this COVID-19. Like in our hospitals, drugs are not available. We heard that most of the drugs come from abroad like India and other countries and airplanes are not coming as such it is difficult to get drugs in our hospitals.” (FGD_Balaka Standard 6 Boys).

#### Shift in focus from malaria to COVID-19

The parents also added that health providers have shifted their focus and effort from prevention and treatment of malaria to COVID-19. They explained that most activities intended for malaria control were suspended. For example, the outreach and village clinics for treating malaria cases in the community were no longer happening.

"It has affected us greatly in the sense that HSAs are not coming for outreach services because of the restrictions on staying in groups. They have stopped educating us about how to prevent and treat malaria as more focus is now centered on COVID-19 than malaria." (FGD_Machinga Female Parent of SAC).

#### Fear of contracting and testing for COVID-19 at the facility

Furthermore, the fear of contracting COVID-19 at the health facility discouraged community members from seeking treatment from the facilities. Community leaders reported that people were reluctant to visit the health centres for fear of testing positive for COVID-19, particularly those experiencing symptoms like headache and fever as narrated below:

"Most people are now discouraged to go and seeking malaria treatment at the hospital because of fears and worries that they can contract the disease [COVID-19] there. Some people could just stay at home even if they feel malaria signs and this might, in turn, lead to untimely deaths from malaria." (KII_Machinga faith leader).

"Many people were afraid to go to the hospital for fear that they might test positive for COVID-19…because by telling them that you have signs like sores on the throat, people were afraid. There were several people we [Community Leaders] talked to on the same issue. You might find someone in tears and ask them what the problem is…you would hear them say that they have pains in the chest and ribs; if you ask them why they were not going to the hospital, you would hear them say I am afraid that I might be COVID-19 positive”. (KII_Balaka Community Leader).

## Discussion

The WHO recommends malaria diagnosis and effective treatment as one of the key strategies for malaria control and eradication [1, 22, 23]. The findings from this study highlight the complexity of providing access to prompt and effective malaria treatment. While access can be considered as simply the freedom to use health services, various barriers linked to providers, individuals and wider institutional factors are all intertwined to determine whether or not services are actually present, available, acceptable and used [24]. Thiede et al*.* [25] characterized access as a multidimensional concept consisting of affordability, acceptability and availability of services. Affordability relates to the ability of users to cover healthcare costs; acceptability involves beliefs and perceptions about treatment effectiveness and trust in the healthcare system; and availability extends beyond proximity to the facilities to include the availability of resources and opening hours [26]. The findings of this study reaffirm these dimensions of access by bringing to light the barriers that hinder SAC from accessing malaria treatment.

Health system factors that are determined by government policy and action, play a significant role in the accessibility of healthcare services. Findings from the current study suggest that long queues, due to high patient volume and limited healthcare workers, result in longer waiting time and prolonged stays at the facilities. This discourages SAC from visiting health facilities to access malaria treatment, as doing so also affects their education. In Malawi, people of all ages and disease severity use the same queue for outpatient services, which has previously resulted in delays in treating seriously ill under-five children [19]. Similarly, a study conducted in Sierra Leone attributed long wait times to high demand from people seeking services in the health facilities [27]. Thus, those seeking care opt to not join or to leave the queue, and not access the services [28], suggesting the importance of reducing wait times. To address this issue, interventions such as increasing the number of healthcare providers have been suggested to reduce pressure on the entire team [29–31]. This could allow implementation of multiple-server queueing models that have proven to reduce patient's wait time at health facilities [31–33].

One of the perennial problems affecting access to treatment of malaria is the frequent stock-outs of essential supplies, including anti-malarial drugs and malaria testing kits. This is commonly observed in rural health facilities in Malawi [34], as well as in other parts of the malaria endemic region [24, 35]. In Malawi, people are denied treatment sometimes when RDTs are depleted, because guidelines require that anti-malarials only be given upon a positive malaria test. [36]. Such stock-outs discourage families from visiting health facilities, leading them to seek alternative options or incur financial burdens from local private pharmacies [37]. In Malawi, poor record keeping results in poor demand forecasting which negatively affects the supply chain [36], leading to stockouts of key commodities [38]. An earlier study also reported that inadequate financial resources and an inability to properly forecast need contributed to stock-outs in an integrated community case management (iCCM) programme [39, 40]. Efforts to improve stock management, such as enhancing record-keeping practices and addressing financial constraints, are crucial.

Long distances to health facilities pose a significant obstacle for SAC seeking malaria treatment. This challenge is particularly prevalent in rural areas where transportation costs are often higher. Distance has been reported as a barrier by studies in Malawi [41], Uganda [42, 43], Tanzania [44, 45], Equatorial Guinea [46], and Ethiopia [47]. Consequently, SAC often resort to obtaining treatment from untrained local vendors or private facilities [48], leading to inappropriate or incorrect malaria treatment [49–51]. Previous research in this context has shown that SAC were more likely to seek malaria treatment from informal providers as compared to other age groups [7, 12], as a consequence of these access barriers. To address this, Malawi should consider expanding the age range of children who are served at its village clinics or introduce school-based access to malaria treatment. Village clinics, as part of iCCM, have been successfully implemented in providing treatment for common childhood diseases for under-five children in Malawi [40].

Provider attitudes play a significant role in hindering access to health services, as revealed by this study. Participants commonly reported that poor provider attitudes discouraged them from going to facilities, even when the facility was nearby. Provider attitudes affect people’s satisfaction and trust in the health services being provided, thereby influencing whether they actually use those services [24]. Similar findings have been reported in other low income settings where patients felt neglected or mistreated by healthcare providers [42, 52]. The other barrier reported from the current study is the limited operating hours of health facilities, which coincide with school hours, making it difficult for SAC to access malaria treatment. In rural Malawi, most facilities open in the morning, but are closed in the afternoon. To improve access by SAC, deliberate efforts should be made to educate providers on how to provide child-friendly services and expand service times to accommodate the needs of school children. As suggested by prior studies in Malawi [53, 54], schools could also consider the introduction of school clinics run by either teachers or health professionals to provide in-house malaria treatment for children.

Fear of being tested for malaria emerged as a barrier to accessing malaria treatment in the study. Concerns about pain, infection during a finger prick, and the possibility of blood samples being tested for other diseases other than malaria highlights the importance of addressing personal knowledge and cultural beliefs to improve access to treatment. A study conducted in Tanzania [55] suggested that use of RDTs was more accepted when test results were positive, as that represented confirmation of the presence of malaria parasites, and allowed for appropriate counselling and treatment. This suggests that effective health education and information would help increase acceptability of, hence access to, malaria testing and treatment. Such interactions, either as part of the school curriculum or with health workers, would also educate SAC about the true causes of malaria, which unfortunately is sometimes still considered related to witchcraft [56].

The COVID-19 pandemic disrupted routine health services and diverted already limited resources from critical diseases like malaria. This study found that SAC were significantly affected by COVID-19 due to reduced health services and access restrictions to health facilities. Families were also reluctant to visit the facilities for fear of contracting COVID-19 there, and/or being tested for it, as symptoms can be similar to those of malaria. Similar findings were observed in other studies conducted in sub-Saharan Africa where people stopped accessing services at health facilities for fear of contracting COVID-19 [57], as well as fear of testing positive to COVID-19 [58–60]. The shift in focus to COVID-19 further decreased malaria control activities. Studies conducted during the COVID-19 period confirmed COVID-19 reduced the time devoted to managing other diseases, and people stopped going to the facilities [58], as well as reducing the information that people received about other diseases such as malaria [57].

An analysis of routine surveillance data comparing monthly malaria cases during January to June of 2017–2020 in Zimbabwe revealed that there was an increase in malaria incidence when COVID-19 hit the nation [61]. Simulation modelling studies also predicted increased malaria morbidity and mortality in SSA due to COVID-19's impact on malaria control efforts [62–64]. Although COVID-19 service provision and messaging were important, integrating its messages with those of other diseases is crucial for resource efficiency. There is also a need to ensure continued provision of malaria treatment and control services during COVID-19 pandemic to sustain progress and prevent setbacks in the fight against malaria.

## Conclusions

This study identified numerous health system barriers to accessing malaria treatment for SAC in Malawi, including common factors that are cited by the general population. This highlights the WHO recommendation that health systems across malaria endemic regions need to be strengthened if malaria eradication is to be achieved. In addition, the study highlighted the negative impact of COVID-19 on SAC’s. Urgent interventions are needed to improve SAC’s access to prompt diagnosis and treatment, such as expanding the age range of children receiving treatment at village clinics and establishing school clinics for in-house malaria treatment. These measures are needed for effectively manage malaria in school aged children.

## Data Availability

The dataset analysed for this study is available from the corresponding author on reasonable request.

## Abbreviations

ACT: Artemisinin-based combination therapy
COVID-19: Coronavirus Disease 2019
DHMT: District Health Management Team
FGD: Focus group discussions
FL: Free listing interviews
iCCM: Integrated Community Case Management
ICEMR: International Center of Excellence in Malaria Research
IPTp: Intermittent preventive treatment in pregnancy
IRS: Indoor residual spraying
KII: Key Informant Interviews
LLIN: Long-Lasting Insecticidal Net
RDT: Malaria Rapid Diagnostic Test
NMCP: National Malaria Control Programme
SAC: School-age children
SSA: Sub-Saharan Africa
SSI: Semi-structured interviews
